# Effect of AST-120 on Endothelial Dysfunction in Adenine-Induced Uremic Rats

**DOI:** 10.1155/2014/164125

**Published:** 2014-04-14

**Authors:** Yuko Inami, Chieko Hamada, Takuya Seto, Yoko Hotta, Seiki Aruga, Jiro Inuma, Kosuke Azuma, Hiroaki Io, Kayo Kaneko, Hirotaka Watada, Yasuhiko Tomino

**Affiliations:** ^1^Division of Nephrology, Department of Internal Medicine, Juntendo University, Faculty of Medicine, 2-1-1 Hongo, Bunkyo-ku, Tokyo 113-8421, Japan; ^2^Division of Metabolism and Endocrinology, Department of Internal Medicine, Juntendo University, Faculty of Medicine, Tokyo, Japan

## Abstract

*Aim.* Chronic kidney disease (CKD) represents endothelial dysfunction. Monocyte adhesion is recognized as the initial step of arteriosclerosis. Indoxyl sulfate (IS) is considered to be a risk factor for arteriosclerosis in CKD. Oral adsorbent AST-120 retards deterioration of renal function, reducing accumulation of IS. In the present study, we determined the monocyte adhesion in the adenine-induced uremic rats *in vivo* and effects of AST-120 on the adhesion molecules. *Methods*. Twenty-four rats were divided into control, control+AST-120, adenine, and adenine+AST-120 groups. The number of monocytes adherent to the endothelium of thoracic aorta by imaging the entire endothelial surface and the mRNA expressions of adhesion and atherosclerosis-related molecules were examined on day 49. The mRNA expressions of ICAM-1 and VCAM-1 in human umbilical vein endothelial cells were also examined. *Results*. Adenine increased the number of adherent monocytes, and AST-120 suppressed the increase. The monocyte adhesion was related to serum creatinine and IS in sera. Overexpression of VCAM-1 and TGF-**β**1 mRNA in the arterial walls was observed in uremic rats. IS induced increase of the ICAM-1 and VCAM-1 mRNA expressions *in vitro*. *Conclusion*. It appears that uremic condition introduces the monocyte adhesion to arterial wall and AST-120 might inhibit increasing of the monocyte adherence with CKD progression.

## 1. Introduction

Chronic kidney disease (CKD) is an independent risk factor for development of cardiovascular disease (CVD) and increased morbidity and mortality associated with the disease [1]. An American Heart Association statement in 2003 recommended that patients with CKD should be considered as part of the highest risk group for subsequent CVD events [2]. Several reports have shown that not only end-stage kidney disease (ESKD) but also mild renal dysfunction is cardiovascular risk factors [3, 4]. Details of interactions between the kidneys and the cardiovascular system remain unclear. Endothelial dysfunction is an initial step of atherosclerosis in patients with risk factors such as hypertension, diabetes, and CKD [5, 6]. Endothelial dysfunction may play an important role in the development of CVD [7], which is a leading cause of mortality in CKD patients [8]. Progressive deterioration of renal function in CKD patients may lead to accumulation of uremic toxins, which may induce production of free radicals and activate proinflammatory factors, leading to vascular endothelial cell dysfunction and injury, followed by development of CVD. The role of uremic toxins in the development of CVD in CKD patients is not well known.

Indoxyl sulfate (IS) is a uremic toxin that accelerates the development of CKD [9]. Serum levels of IS are elevated in patients with CKD and appear to be correlated with CKD progression. Several researchers have reported that IS could play a role in the endothelial dysfunction in uremic patients [10, 11]. Dou et al. [11] showed that IS, a protein-bound uremic solute, induces endothelial dysfunction by inhibiting endothelial proliferation and migration* in vitro*. AST-120 (Daiichi-Sankyo Industry Co., Tokyo, Japan) is an oral charcoal adsorbent that reduces the levels of circulating uremic toxins such as IS and indole acetic acid. AST-120 prevents the progression of renal insufficiency through the reduction of uremic toxins [10, 12, 13]. Several reports have demonstrated that AST-120 potentially prevents histological and functional aggravation of CKD in humans and an animal model of CKD [12–14]. Thus, IS is involved in the development of CVD. Recently, Azuma et al. [15, 16] established a new en face method for optimal observation of the endothelial surface (NEMOes), which is suitable for quantification of the numbers of monocytes that adhere to rat thoracic aorta after immunostaining of the monocyte/macrophage specific protein, CD68.

We hypothesized endothelial dysfunction associated with elevation of IS in uremic condition involved in stimulating monocyte adherence to arterial wall similar to the first step of atherosclerosis. And AST-120, an absorbance of IS, may suppress the monocyte adherent via inhibition of adhesion molecules expressions. The objective of the present study was to evaluate the effect of AST-120 on the endothelial dysfunction using NEMOes in adenine-induced uremic rats.

## 2. Materials and Methods

### 2.1. Reagents

Human umbilical vein endothelial cells (HUVECs) and Clonetics EGM-2 BulletKit (CC-3162) were purchased from Sanko Junyaku Co. (Tokyo, Japan). AST-120 was synthesized by and kindly provided by Daiichi-Sankyo Pharmaceuticals Co. Ltd. (Tokyo, Japan). Adenine and IS were obtained from Sigma Chemical Co. (St. Louis, MO, USA). All other chemicals were purchased from Wako Pure Chemical Industries (Osaka, Japan). RNeasy Mini Kit and RNase-Free DNase Set were obtained from Qiagen (Courtaboeuf, France). The StrataScript First-Strand Synthesis System and Full Velocity SYBR Green qPCR Master Mix were obtained from Stratagene Europe (Amsterdam, the Netherlands). All primers were purchased from Invitrogen (CA, USA).

### 2.2. Animals

Male Sprague-Dawley rats, 8 weeks of age, obtained from Charles River Japan (Yokohama, Japan) were fed a standard powder diet containing 1.02% phosphorus, 1.08% calcium, 25.1% crude protein, and 2.4 IU/g vitamin D_3_ (CE-2; CLEA Japan, Japan) for 11 days pretreatment as an acclimatization period. Rats were kept in cages (three or four animals in each cage) and allowed free access to food and water. The animal room was kept on a 12/12 hour light/dark cycle (7:00 am to 7:00 pm dark, 7:00 pm to 7:00 am light), at a constant temperature of 22 ± 1°C and relative humidity of 55 ± 5% throughout the experimental period. The study protocol was reviewed and approved by the Animal Care and Use Committee of Juntendo University.

### 2.3. Experimental Design (Figure 1(a))

Twenty-four rats were prepared in this study. Twelve rats were fed standard powder diet and other twelve rats were fed 0.75% adenine-containing powder diet for 3 weeks as the first stage of treatment. Then the rats were divided into 2 groups, the rats treated with AST-120 and the control rats. The AST-120 treated rats were fed a standard powder diet with 5% AST-120 for 4 weeks as the second stage of treatment. Finally, the 24 rats were divided into 4 groups of six animals each (control group, adenine group, control + AST-120 group, and adenine + AST-120 group). After the second stage of treatment, all rats were immediately sacrificed by heart puncture under ether anesthesia according to the Animal Care and Use Committee of Juntendo University. Body weight and blood pressure were measured on days 0, 21, and 49. Systolic arterial blood pressure (SBP) was measured by the tail-cuff method on prewarmed rats (BP-98A; Softron, Tokyo, Japan). Blood samples were taken on days 0, 21, and 49 in each group from the tail vein and by heart puncture under ether anesthesia on day 49. Levels of serum phosphorus, calcium, albumin, creatinine, urea nitrogen (UN), c-reactive protein (CRP), intact parathyroid hormone (iPTH), indoxyl sulfate (IS), and pentosidine were examined by a contract laboratory (SRL Co., Tachikawa, Japan). Plasma indoxyl sulfate (IS) [17] and pentosidine [18] were measured by high-performance liquid chromatography (HPLC).

### 2.4. New En Face Method for Optimal Observation of the Endothelial Surface (NEMOes)

**Figure 1:**
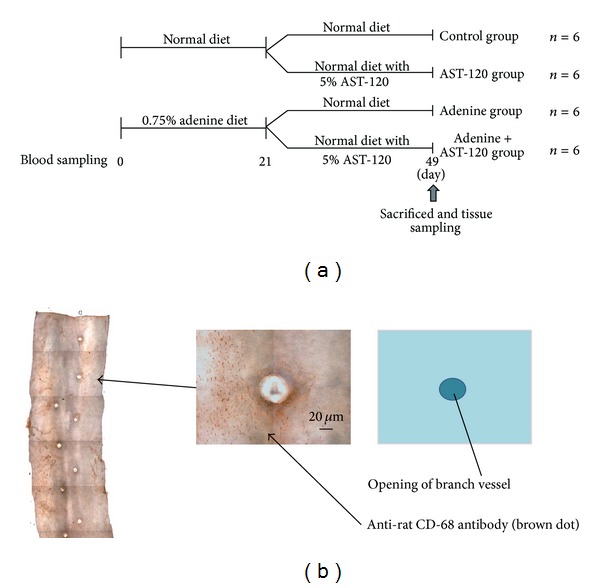
(a) Experimental design and (b) assessment of a new en face method for optimal observation of endothelial surface (NEMOes) number of adherent mononuclear cells.

Monocyte adhesion to the wall of the thoracic aorta in rats was examined by NEMOes as described previously [15, 16]. Briefly, rats were sacrificed and perfused with normal saline followed by 10% buffered formalin into aorta. After fixation, the aorta was divided into segments 8–12 mm long. Each segment was then placed in 0.05% hydrogen peroxidase, then incubated with mouse anti-rat CD68 antibody (Serotec, Raleigh, NC, USA), and diluted 1 : 100 in phosphate-buffered saline (PBS). After the staining, the segments were cut open longitudinally along the ventral side with scissors. Specimens were viewed under a microscope (E800; Nikon, Tokyo, Japan) connected to an* XYZ* controller and a digital camera (Media Cybernetics Inc., Silver Spring, MD, USA). Pictures were taken at various focal lengths with an automatically regulated* Z*-stepper and the clearest images were selected automatically to produce a composite image of the whole thoracic aorta by Image-Pro4.5 J (Planetron, Tokyo, Japan) [16]. To quantitate the exact number of monocytes adhering to the endothelium, we counted separately the numbers of CD68-positive tear-shaped cells around the openings of intracostal arteries in each aorta (1400 *μ*m × 1000 *μ*m) (Figure 1(b)). The cell density in each area was calculated as the cell count divided by the total area by examiners blinded to the treatment regimen.

### 2.5. Cell Culture

HUVECs were seeded on the gelatin-coated culture plates and cultured in EGM-2 medium (Takara Bio Inc., Shiga, Japan) containing 0.5% fetal bovine serum rum (FBS, GIBCO, California, USA) under standard cell culture conditions (humidified atmosphere, 5% CO_2_, 37°C). The cells between passages 4 and 6 were used for the experiment. HUVECs were cultured on gelatin-coated culture plates until confluence. HUVECs were incubated with IS at various concentrations (100, 250, and 500 *μ*M) for 24 hours.

### 2.6. Quantitative RT-PCR

Total RNA was extracted from the thoracic aorta of each rat and the HUVECs using RNeasy Mini Kit and RNase-Free DNase Set purchased from Qiagen (Courtaboeuf, France). Then cDNAs were synthesized using dNTP mix and random primers. The resulting cDNAs were amplified using SYBR Green PCR Kit (Applied Biosystems, Foster City, CA, USA). Quantitative PCR was performed on an ABI PRISM 7700 sequence detection system (Perkin Elmer Life Sciences Inc., Boston, MA, USA). The relative abundance of mRNAs was calculated by the comparative cycle of threshold (CT) method with GAPDH mRNA as the invariant control. The primers used in this study are listed in Table 1.

### 2.7. Statistical Analysis

The results are expressed as mean ± SD. Correlations between different parameters were analyzed by the 2-tailed Student's *t*-test or by regression analysis. ANOVA was used to determine differences in the characteristics among multiple groups. Nonparametric methods, such as Spearman's rank correlation and Wilcoxon's rank-sum test were used for correlation and 2-sample comparisons, respectively. *P* < 0.05 was considered as significant. These statistical analyses were performed with Stat View 5.0 software (Abacus Concepts).

## 3. Results

### 3.1. Laboratory Findings

Characteristics and biochemical parameters on day 49 are shown in Table 2. The adenine-treated rats indicated increases of serum urea nitrogen (s-UN) and creatinine (s-Cr) compared with those in control rats. Both average food intake and body weights until day 21 did not differ among the groups (data not shown). AST-120 decreased the elevated IS levels in the adenine-treated rats. Increments of s-UN and s-Cr were not significantly suppressed by AST-120 in the uremic rats. AST-120 also decreased serum Pi levels, but the difference was not statistically significant.

### 3.2. Monocytes Adherent to Endothelium* In Vivo*

**Figure 2:**
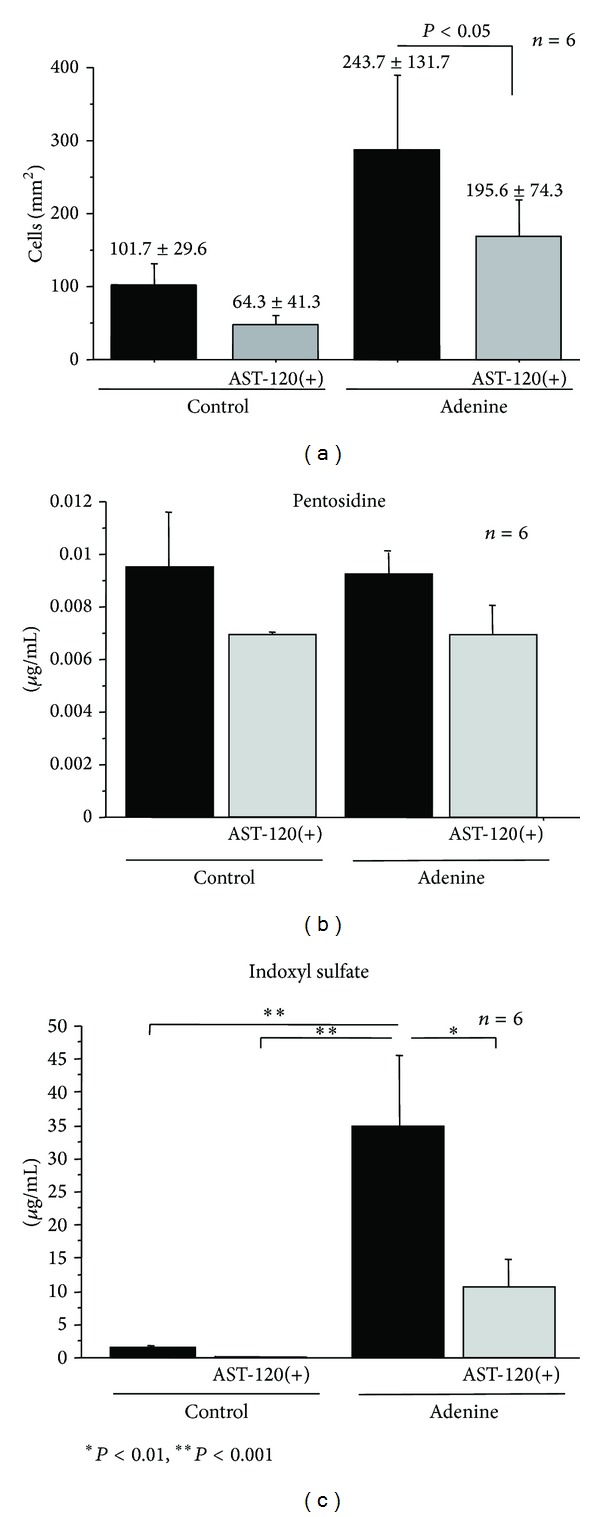
(a) Monocyte adhesion to the endothelium assessed by NEMOes, (b) levels of pentosidine in sera, and (c) levels of indoxyl sulfate in sera *P < 0.01 and **P < 0.001.

The number of adherent CD-68 positive cells to endothelium was increased in the adenine-treated uremic rats on day 49 (Figure 2(a)). AST-120 suppressed the increase of monocyte adhesion induced by adenine (195.6 ± 74.3 versus 243.7 ± 131.7, resp., *P* < 0.05, Figure 2(a)). Adenine increased in rat serum IS levels but not in pentosidine levels (Figures 2(b) and 2(c)). AST-120 significantly suppressed the IS elevation in the uremic rats (Figure 2(c)).

### 3.3. Relationship between the Number of Monocytes Adherent to Endothelial Cells (NMAE) and Levels of Serum Cr and IS

**Figure 3:**
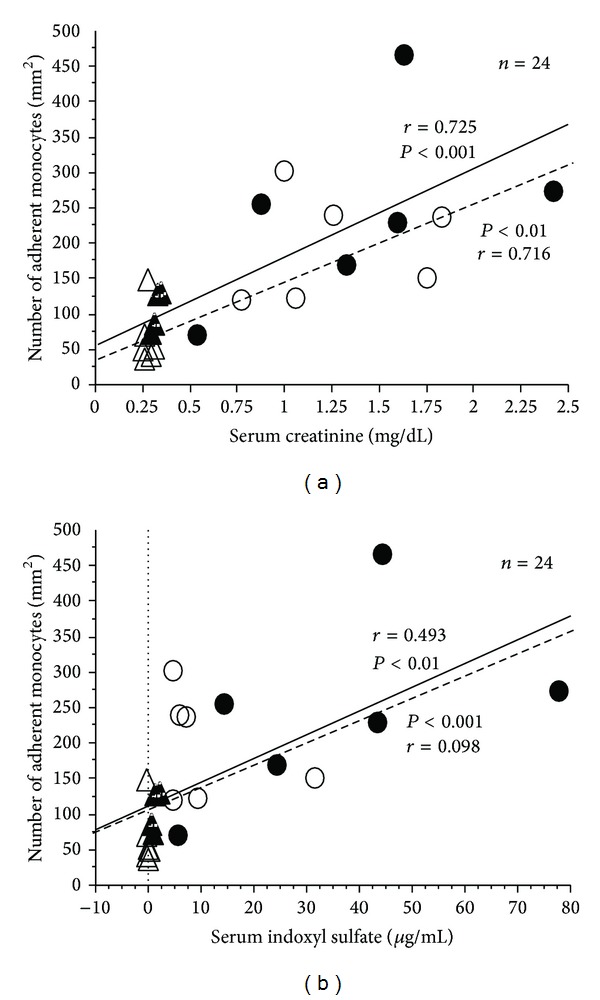
Relationship between the number of monocytes adherent to endothelial cells and the level of serum creatinine (Cr) and indoxyl sulfate (IS). Closed circles: adenine alone; open circles: adenine + AST-120; closed triangles: control; open triangles: control + AST-120. Solid line: the control and the adenine rats (*n* = 12); dotted line: the control + AST-120 and the adenine + AST-120 rats (*n* = 12).

The number of monocytes adherent to the endothelium was significantly related to the level of s-Cr (Figure 3(a)) and IS in sera (Figure 3(b)), respectively. No significant suppressive effect in the monocyte adherent was observed in both s-Cr and IS.

### 3.4. Expressions of Adhesion and Atherosclerosis-Related Molecules in the Thoracic Aorta

**Figure 4:**
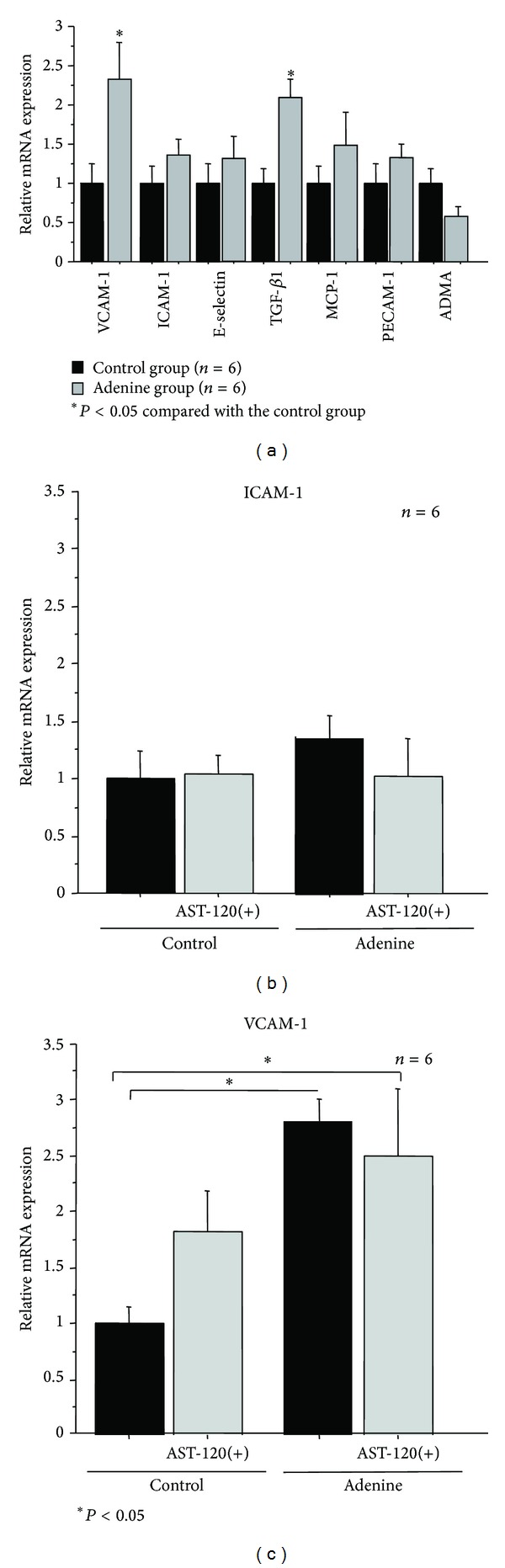
Expression of adhesion molecules, growth factors, and endothelial dysfunction factor. Levels of ICAM-1, VCAM-1, TGF-*β*1, MCP-1, E-selection, PECAM-1, and ADMA mRNA in thoracic aorta were determined in the control and adenine rats at 7 weeks. (b) and (c): expressions of ICAM-1 and VCAM-1 mRNA in thoracic aorta. Quantitative RT-PCR of ICAM-1 and VCAM-1 using RNA extracted from the thoracic aorta in the control, control + AST-120, adenine, and adenine + AST-120 groups (*n* = 6). The relative mRNA level was calculated with the level of the control group set at 1. Data are expressed as mean ± SD of five rats in each group.

Expressions of VCAM-1, ICAM-1, E-selectin, TGF-*β*1, MCP-1, PECAM-1, and ADMA mRNA extracted from the thoracic aorta were measured in the groups at 7 weeks to investigate the effect of AST-120 on the synthesis of adhesion molecules. Many mRNA expressions of adherent and atherosclerosis-related factors were increased but not statistically significant except VCAM-1 and transforming growth factor-beta 1 (TGF-*β*1) in the adenine rats (Figure 4(a)). No significant difference was observed in level of ICAM-1 expression (Figure 4(b)). The levels of VCAM-1 mRNA expression in the adenine and adenine + AST-120 group were higher than those in the control group (Figure 4(c)). AST-120 did not influence the synthesis of adhesion molecule mRNA expressions in both the control and AST-120 groups (Figures 4(b) and 4(c)).

### 3.5. Effect of IS in HUVECs Expression of ICAM-1 and VCAM-1 mRNA* In Vitro*

**Figure 5:**
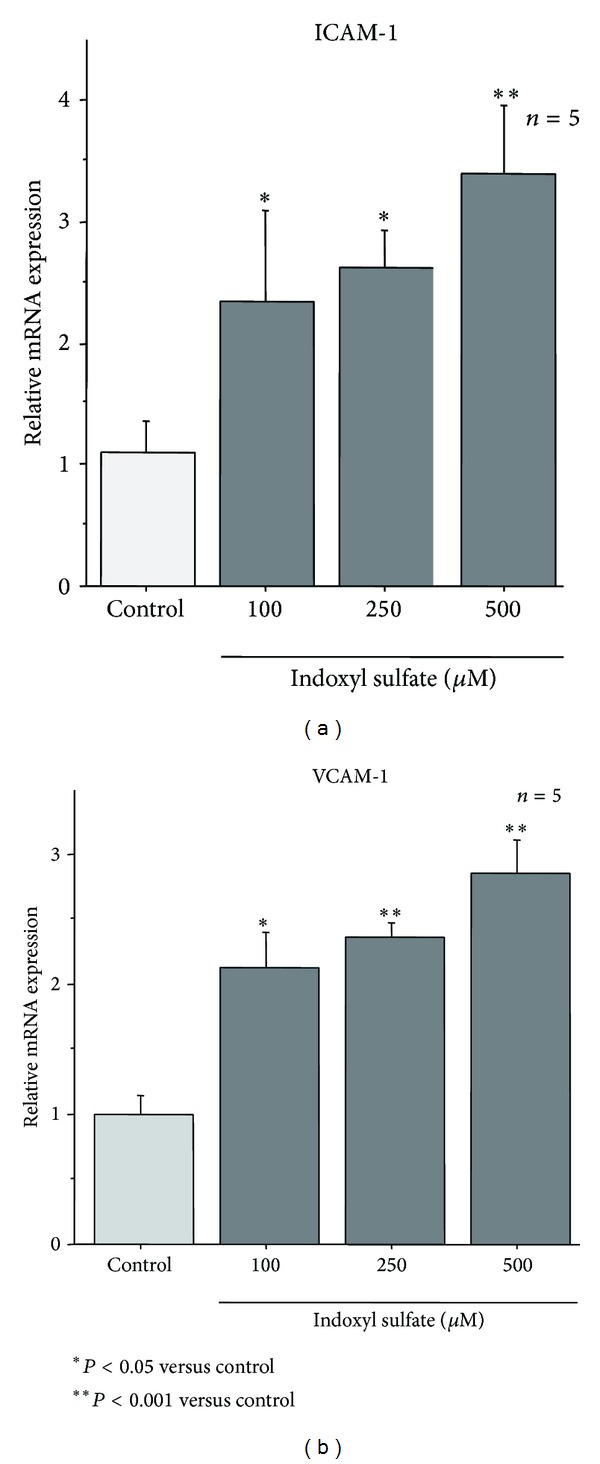
Expression of ICAM-1 and VCAM-1 mRNA in HUVECs. Expressions of adherence factors; ICAM-1 and VCAM-1 mRNA were examined to clarify the effect of IS on synthesis of HUVECs* in vitro* (*n* = 5). Both syntheses of ICAM-1 and VCAM-1 mRNA were accelerated by IS in dose-dependent manner. *P < 0.05 compared to the control group. **P < 0.001 compared to the control group.

To evaluate IS effect on expression of adherence molecules, that is, ICAM-1 and VCAM-1, we measured these mRNA expressions of HUVECs cultured in medium containing various concentrations of IS for 24 hours. Both syntheses of ICAM-1 and VCAM-1 mRNA were accelerated by IS in a dose-dependent manner (Figures 5(a) and 5(b)). At these concentrations, IS neither affected endothelial viability nor induced apoptosis (data not shown).

## 4. Discussion

It is well known that CKD is an independent risk factor for development of atherosclerosis, especially ischemic heart disease. Aggressive and systematic therapeutic strategies are considered to ameliorate high mortality in CKD patients [1, 2, 7, 8]. It has been suggested that atherosclerosis might be developed at early stage of CKD and accumulation of uremic toxins might play an important role in the progression [3, 4], but the relationship between atherosclerotic changes and action of uremic toxins in early stage of CKD is unclear. In the present study, we obtained pathologic findings of developing atherosclerosis in early stage of CKD* in vivo*, and we evaluated whether NEMOes can be used for assessment of endothelial dysfunction in CKD.

An oral adsorbent, AST-120, is effective in removing uremic toxins including indoxyl sulfate and can delay the progression of chronic renal failure and the need for dialysis in uremic patients [13, 19, 20]. In molecular basis of renal inflammation and fibrosis, AST-120 significantly reduced renal expression of ICAM-1, osteopontin, monocyte chemotactic protein (MCP-1), and TGF-*β*1 in diabetic rats [12, 21]. According to these reports, we determined that the preventive effect of AST-120 on the development of atherosclerosis in early stage of CKD was examined in adenine-induced uremic rats. This study revealed that the number of adherent mononuclear cells was increased in uremic condition and indicated significant relation with IS and creatinine in sera. Uremic condition stimulated TGF-*β*1 and VCAM-1 expressions* in vitro*. AST-120 significantly suppresses the monocyte adherence without significant VCAM-1 overexpression in uremic rats.

Proinflammatory cytokines, hypoxia, and uremic toxin induced endothelial dysfunction in CKD [22, 23]. A widely popular definition of the endothelial dysfunction includes overexpressing adhesion molecules such as ICAM-1, VCAM-1, E-selectin, and P-selectin in the vascular endothelium as well as physiological changes such as flow-mediated vasodilatation (FMD) or forearm blood flow (FBF). To examine atherosclerotic changes in the early stage of CKD, we examined endothelial dysfunction in younger rats, treated with adenine only for 3 weeks, which was a relatively shorter period than in the previous studies [24], and not given high phosphate diet. According to the results, IS may play a role in developing atherosclerosis in CKD. Motojima el al. [25] reported that elevation of ICAM-1, but not VCAM-1 mRNA, was observed in monocyte adhesion in type 2 DM rats fed a high-fat diet. And they reported that the fluctuation in blood glucose enhanced monocyte adhesion to the endothelium in diabetes rats using the NEMOes. They also pointed that there was no significant increase in the expressions of either ICAM-1 or VCAM-1. In our study, no significant difference between the uremic rats and the control rats was observed in the levels of E-selectin, PECAM-1, and ADMA. As mentioned above, NEMOes appears to be a highly sensitive evaluation method for endothelial dysfunction under uremic conditions. It is limitations of our study to clarify the relationship between the monocyte adherence and the uremic toxins, since the number of subjects was small. Since AST-120 suppressed the monocyte adherent, the suppressive effect of AST-120 on other uremic toxins as well as indoxyl sulfate (IS) should be examined in the future. We hypothesized that the monocyte adhesion is not only dependent on the overexpression of adhesion molecules but also circulating monocyte dysfunction and other factors.

Recent reports suggested that AST-120 attenuated the development of glomerular sclerosis and interstitial fibrosis through suppression of oxidative stress and fibronectin expression in experimental diabetes rats [26, 27]. Nakamura et al. [28] reported that AST-120 decreased carotid intima-media thickness and arterial stiffness in nondiabetic CKD patients. This study revealed that AST-120 delayed the increase of the adherent monocyte with developing uremic condition. Since IS stimulated synthesis of ICAM-1 and VCAM-1 mRNAs in HUVECs dose-dependently, it was suggested that IS might play a role in acceleration of monocyte adhesion in the uremic condition according to increasing production of adhesion molecules in the endothelial cells. Since we presented the elevation of TGF-*β*1 mRNA expression in the uremic rats, uremic condition may induce median calcification, a characteristic of atherosclerotic morphologic changes in CKD. It is important to examine the relationship between monocyte dysfunction and atherosclerosis in the future.

It appears that uremic condition introduces the monocyte adhesion to arterial wall and AST-120 might inhibit increasing of the monocyte adherence with CKD progression.

## Figures and Tables

**Table 1:** RT-PCR primers for rat thoracic aorta.

GAPDH	L	TGCACCACCAACTGCTTAG
	R	GGATGCAGGGATGATGTTC

TGF-*β*1	L	AGTCCCAAACGTCGAGGTGA
	R	AGGTGTTGAGCCCTTTCCAG

VCAM-1	L	CAAGGCTACATGAGGGTGCT
	R	TAAGGTGAGGGTGGCATTTC

ICAM-1	L	TTTGAGGAAAGCACCCTGAC
	R	ATTGCCTAGACCCTGGTGAA

MCP-1	L	CAGATCTCTCTTCTCCACCACTAT
	R	ACAGGCAGCAACTGTGAACAA

E-selectin	L	CCTCGTGCTTTCTCTCTGCT
	R	ATCGCCACCAGATGTGTGTA

PECAM-1	L	CCCCAGTTCCACTTTTTCAA
	R	AGGTGACCGTGGACAAAAAG

ADMA	L	ACCCTGTCTACGTGCAGTCC
	R	TGCTAATGGGAAACCCTGTC

**Table 2:** Comparison of characteristics and biochemical parameters.

Parameters	Control	Control + AST-120	Adenine	Adenine + AST-120
	(*n* = 6)	(*n* = 6)	(*n* = 6)	(*n* = 6)
BW (g)	458.5 ± 17.2	429.3 ± 20.5	309.5 ± 69.2^a,b^	322.4 ± 51.3^a,b^
SBP (mmHg)	131.00 ± 6.06	122.00 ± 5.57	136.6 ± 21.90^a^	122.8 ± 9.27
S-UN (mg/dL)	25.00 ± 3.89	25.08 ± 7.65	113.52 ± 62.34^a,b^	102.72 ± 34.41^a,b^
S-Cr (mg/dL)	0.330 ± 0.026	0.284 ± 0.025	1.572 ± 0.561^a,b^	1.334 ± 0.452^a,b^
Ca (mg/dL)	11.33 ± 0.98	11.48 ± 0.70	10.90 ± 1.35	11.40 ± 1.16
Pi (mg/dL)	11.33 ± 2.72	10.48 ± 0.88	13.04 ± 4.58	12.58 ± 1.33
Alb (g/dL)	4.38 ± 0.22	4.34 ± 0.15^c^	4.12 ± 0.49	3.80 ± 0.28^c^
iPTH (pg/mL)	<3	<3	<3	<3
CRP (mg/dL)	0.010 ± 0.00	0.010 ± 0.00	0.028 ± 0.040	0.012 ± 0.004
Indoxyl sulfate (*μ*g/mL)	1.61 ± 0.64	0.27 ± 0.19	40.82 ± 24.33^a,b^	11.76 ± 11.14^d^

All data are given as mean ± SD.

^a^ *P* < 0.01 versus control, ^b^ *P* < 0.01 versus control + AST-120, ^c^ *P* < 0.05 versus control, and ^d^ *P* < 0.01 versus adenine.

Abbreviations: SBP: systolic blood pressure; S-UN: serum urea nitrogen; S-Cr: serum creatinine; Ca: calcium; Pi: phosphorus; Alb: albumin; iPTH: intact parathyroid hormone; CRP: c-reactive protein.
